# An Investigation into the Footing Profile Suppression in (110) Si Anisotropic Etching

**DOI:** 10.3390/mi17050518

**Published:** 2026-04-24

**Authors:** Zhishen Wang, Guoliang Xie, Gaowei Xu, Genzi Li, Weihu Zhou, Dongzhi Fu, Lingde Kong, Zhiwen Chen, Sheng Liu

**Affiliations:** 1 School of Power and Mechanical Engineering, Wuhan University, Wuhan 430072, China; 2 China Wafer Level CSP Co., Ltd., Suzhou 215026, China; 3 Shanghai Institute of Microsystem and Information Technology, Shanghai 200050, China; xugw@mail.sim.ac.cn; 4 China Productivity Center for Machinery Co., Ltd., Beijing Key Laboratory of Reliability Technology for High-End MEMS Devices, Beijing 100080, China; 5 Institute of Microelectronics, Chinese Academy of Sciences, Beijing 100029, China; 6 Huatian Technology (Kunshan) Electronics Co., Ltd., Suzhou 215300, China; 7 Kunming Institute of Physics, Kunming 650223, China; 8 School of Integrated Circuits, Wuhan University, Wuhan 430072, China

**Keywords:** (110) Si, anisotropic wet etching, V-shaped footing profile, ALE simulation

## Abstract

Deep Si trenches with vertical sidewalls are critical structures in advanced MEMS sensors and microfluidic devices. (110)-oriented Si is specifically required for this purpose, as its crystallographic geometry inherently provides the nearly 90° vertical {111} planes. However, achieving precise morphology on (110) Si remains challenging due to the formation of unwanted V-shaped footing profiles at the bottom. This study establishes a systematically coupled experimental and numerical framework to investigate the anisotropic wet etching mechanism of (110) Si, quantifying the effects of KOH concentration (10–50 wt.%) and temperature (50–90 °C) on profile evolution. Experimental results demonstrate that 10 wt.% KOH at 70 °C yielded the most favorable morphology within the investigated range, with a minimized footing ratio (<2%). Based on these results, a dual-parameter kinetic regulation mechanism is proposed. Low concentration of KOH can minimize the crystallographic etching rate disparity (γ) between fast-etching {100}/{110} and slow-etching {111} planes, while the selected temperature helps maintain interfacial hydrodynamic stability. Furthermore, an Arbitrary Lagrangian-Eulerian (ALE)-based multiphysics model calibrated with Arrhenius kinetics was developed, which captures the overall trend of trench evolution and the dependence of footing formation on KOH concentration and temperature. This work not only provides a recommended process window for suppressing footing defects but also offers a trend-predictive simulation framework for orientation-dependent Si micromachining.

## 1. Introduction

Microelectromechanical systems (MEMS) devices are critical in applications ranging from aerospace to biomedical engineering, with their performance heavily dependent on the precision of Si microfabrication processes [1,2,3]. Among the manufacturing technologies, anisotropic wet etching stands out for its high efficiency, low cost and minimal wafer damage [4,5]. Potassium hydroxide (KOH) solutions are widely used due to their strong crystallographic selectivity, where the {111} plane exhibits an etching rate far lower than those of the {100} or {110} planes [6,7,8,9]. Although KOH etching is a wet-chemical process, it is anisotropic for single-crystal Si because the etch rate strongly depends on crystallographic orientation. While traditional (100)-oriented Si typically produces V-shaped grooves with fixed 54.7° sidewalls, (110)-oriented Si offers nearly 90° vertical sidewalls, owing to the inherent angular relationship between the (110) and {111} planes [10,11]. This characteristic addresses the limitations of (100) Si and makes (110) wafers promising candidates for MEMS devices with high-aspect-ratio features [12,13,14].

However, the practical application of (110) Si is hindered by unresolved process challenges. Unlike the self-limited etching of (100) Si, uncontrolled KOH etching of (110) wafers often introduces severe morphological defects, such as unwanted footing profiles at trench bottoms [15,16]. As elucidated in the comprehensive reviews by Pal and Sato, the stability of etching profiles is fundamentally governed by the crystallographic interplay at corners, where convex corners bounded by fast-etching planes are particularly susceptible to undercutting [17]. This geometric instability extends to the trench bottom in (110) Si, where the convergence of {111} sidewalls can be viewed as a specific manifestation of crystallographic competition similar to corner evolution. These defects degrade pattern fidelity and induce stress concentrations. For instance, V-shaped footing profiles in microfluidic chambers were found to reduce the effective volume, leading to unstable flow and affecting the reproducibility of biochemical reactions. Furthermore, in high-precision MEMS devices, such sharp V-shaped footing profiles can act as stress concentration points and severely compromise the overall structural reliability.

Despite these, systematic research on (110) Si etching remains limited. Early studies mainly focused on feasibility observations or process parameter tuning, such as adding surfactants (e.g., IPA, Triton X-100) [18,19,20,21]. It has been reported that the addition of Triton surfactant to KOH solutions can significantly improve the etching anisotropy and reduce the roughness of {110} planes by forming a selective adsorption layer [22]. Recent reviews by Pal et al. also highlighted that additives like hydroxylamine can dramatically accelerate the anisotropic etching [23]. However, these additives introduce process complexity and potential CMOS incompatibility. Alternative etchants like tetramethylammonium hydroxide (TMAH) can reduce metal contamination but suffered from lower rates and weaker anisotropy [24,25]. Therefore, regulating the intrinsic etching kinetics of pure KOH via concentration and temperature remains the most practical approach. However, precisely identifying the suitable process window traditionally relies on inefficient trial-and-error experiments, because of the lack of a predictive kinetic model that is capable of simulating the dynamic evolution of (110) Si [26]. Although commercial MEMS simulation tools (e.g., IntelliSuite and CoventorWare) can simulate ideal (110) anisotropic etching using empirical etch-rate databases, their etching modules typically rely on purely kinematic models and empirical databases, rather than dynamically coupling local fluid dynamics, mass transport, and surface reaction kinetics. Consequently, they struggle to predict complex kinetic anomalies driven by localized micro-environments, such as the concentration-dependent footing profiles at the bottom of deep trenches, forcing process development to rely on empirical iterations.

To address these challenges, this paper presents a comprehensive study on the suppression of footing profile, a common defect in (110) Si etching. In this work, we propose a kinetic-regulated approach that optimizes intrinsic parameters—specifically KOH concentration and temperature—to balance the trade-off between etching rate and anisotropy. A multiphysics simulation model with Arrhenius kinetics was developed to quantitatively reveal the competition mechanism between the fast-etching {100}/{110} planes and the rate-limiting {111} planes. Through this integrated experimental and simulation framework, we identify the most favorable process window within the investigated conditions for minimizing V-shaped footing profiles.

## 2. Experimental and Simulation Methods

### 2.1. Experimental Setup and Procedures

**Figure 1:**
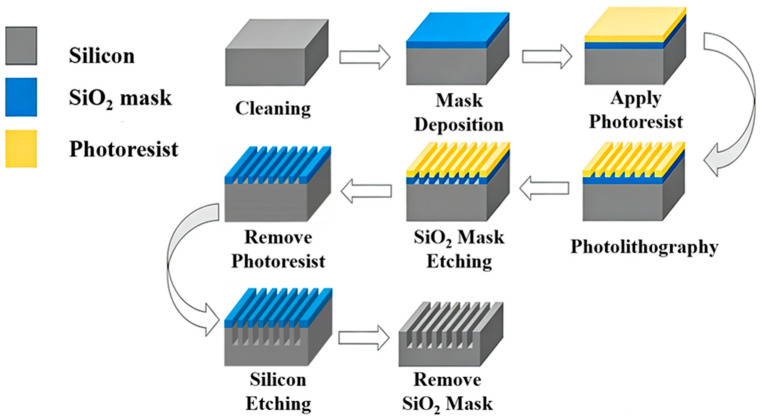
Schematic of the etching test process on (110) Si.

(110)-oriented p-type single-crystal Si wafers were utilized as substrates. As shown in Figure 1, they sequentially underwent ultrasonic cleaning in acetone, ethanol, and deionized (DI) water for 15 min each to eliminate surface contaminants. A 500 nm-thick silicon dioxide (SiO_2_) hard mask was then deposited via plasma-enhanced chemical vapor deposition (PECVD) at 300 °C using a SiH_4_/O_2_ precursor mixture. This thickness was optimized to withstand long-duration etching, given the negligible etching rate of SiO_2_ in KOH (~0.1 nm/min).

Photolithography was performed using AZ4620 photoresist. The trench patterns were defined and transferred to the SiO_2_ layer using a buffered oxide etchant (BOE). The mask edges were precisely aligned with the <111> crystal orientation with an error within ±0.5° to minimize sidewall asymmetry induced by crystallographic misalignment [27].

**Figure 2:**
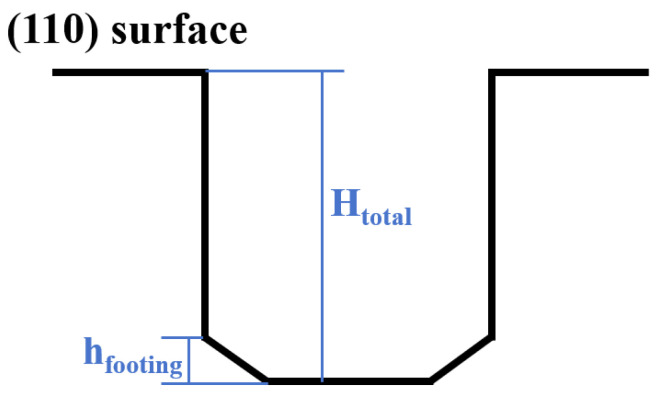
Definition of the footing ratio.

The anisotropic etching was conducted in a thermostatically controlled bath with a temperature accuracy of ±0.5 °C. A magnetic stirrer at 300 r/min was employed to ensure thermal homogeneity and to prevent bubble-shielding defects by accelerating the detachment of the generated H_2_ bubbles during the reaction [28,29]. The etching time t was uniformly set to 30 min. The average etching rate (µm/min) was calculated by dividing the trench depth by the etching duration. The effects of KOH concentrations (10 wt.%~50 wt.%) and temperatures (50 °C~90 °C) on trench morphology were also systematically investigated. After etching, trench morphologies were characterized with scanning electron microscopy (SEM) to quantify relative footing ratio ($R_{footing}$), defined as (Equation (1) and Figure 2).

(1) $$R_{footing}=\frac{h_{footing}}{H_{total}}\times 100\%$$

where $h_{footing}$ represents the vertical height of the tapered region bounded by the inclined {111} facets at the trench bottom, and $H_{total}$ is the total trench depth.

For each etching condition, the experiments were repeated three times, and the trench depth and footing ratio were averaged from these measurements. The footing ratio was calculated from the measured footing height and total trench depth according to Equation (1).

### 2.2. Numerical Simulation

A 3D transient evolution model was developed using COMSOL Multiphysics 6.3 to predict the anisotropic etching behavior of (110) Si. The geometric evolution of the trench was tracked using the Deformed Geometry (dg) interface based on the ALE method.

The chemical basis for this dependence is the reaction between Si and hydroxide ions, which can be described as:

$$Si+2{OH}^{-}+2H_{2}O\to SiO_{2}{\left(OH\right)}_{2}^{2-}+2H_{2}↑$$

This equation highlights that the etching kinetics are governed by the local availability of ${OH}^{-}$ ions, while the generation of hydrogen gas ($H_{2}$) introduces potential bubble-shielding effects [30].

**Figure 3:**
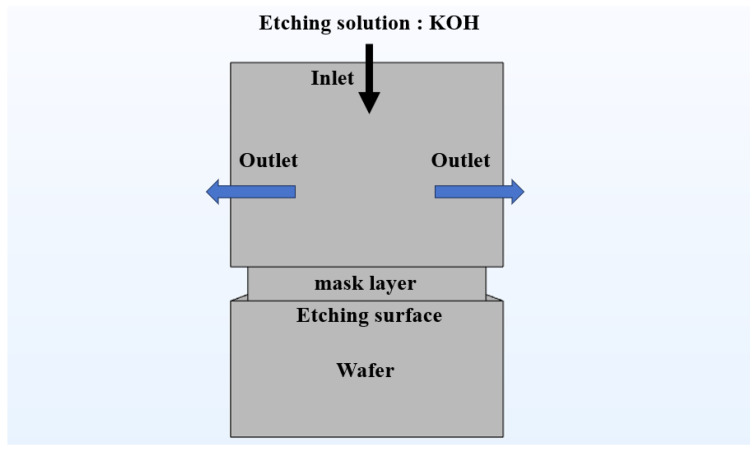
Simulation model of Si wafer etching.

The reaction kinetics of etching were coupled with fluid dynamics and heat transfer in simulations to account for local etchant transport and temperature variations (Figure 3). Specifically, the physical domains were governed by a set of coupled partial differential equations (PDEs). The etchant flow was modeled as an incompressible fluid using the Navier–Stokes and continuity equations:

(2) $$\rho \left(\frac{\partial u}{\partial t}+u\cdot \nabla u\right)=-\nabla p+\mu \nabla^{2}u+F$$

(3) ∇·u=0

where ρ is the fluid density, u is the velocity vector, p is the pressure, and μ is the dynamic viscosity.

The transport of the reactant species (${OH}^{-}$ and $H_{2}O$) was governed by the convection-diffusion equation:

(4) $$\frac{\partial c_{i}}{\partial t}+\nabla \cdot \left(-D_{i}\nabla c_{i}\right)+u\cdot \nabla c_{i}=R_{i}$$

where $c_{i}$ represents the local molar concentration of the species, $D_{i}$ is the diffusion coefficient, and $R_{i}$ denotes the reaction consumption rate at the silicon interface. Furthermore, the local temperature field (T) was resolved by coupling the heat transfer equation:

(5) $$\rho C_{p}\left(\frac{\partial T}{\partial t}+u\cdot \nabla T\right)=\nabla \cdot \left(k\nabla T\right)+Q$$

where $C_{p}$ is the specific heat capacity, k is the thermal conductivity, and Q is the heat source term.

To reproduce the etching process and establish the initial conditions (ICs), the domain at t = 0 was set as a stationary fluid (u = 0) with uniform initial concentration ($c_{i}$ = $C_{0}$) and temperature (T = $T_{0}$). Here, $V_{in}$ was fixed at 0.01 m/s for all simulations. $T_{0}$ was set equal to the prescribed bath temperature of each case, i.e., 50, 60, 70, 80 or 90 °C. $C_{0}$ denotes the inlet etchant concentration corresponding to the selected KOH condition in Table 1, i.e., 10, 20, 30, 40 or 50 wt.%. For the boundary conditions (BCs), the top boundary was defined as the etchant inlet (u = $V_{in}$, $c_{i}$ = $C_{0}$, and T = $T_{0}$), while the lateral boundaries were set as zero-pressure outlets (p = 0) to allow free etchant outflow, as illustrated in Figure 3. At the lateral outlet boundaries, convective outflow conditions were additionally applied for species transport and heat transfer. At the Si–solution interface, the normal recession velocity of the moving boundary was prescribed as:

(6) $$v_{n}=R(\theta ,C,T)$$

where R(θ, C, T) is the orientation-, concentration-, and temperature-dependent etching rate given by the modified Arrhenius model. To resolve the sharp geometric features at the trench bottom, a dynamic mesh refinement strategy was applied at the moving boundary. Critically, this interfacial velocity was implemented in the ALE framework as the prescribed normal mesh velocity at the moving boundary and simultaneously acted as the mass sink for the chemical reactants. This multiphysics model enables the quantitative prediction of V-shaped footing profile formation mechanisms under varying process conditions. To systematically investigate the morphological evolution, 25 simulation cases were conducted by varying KOH concentration and temperature, while keeping the inlet velocity and outlet pressure constant, as summarized in Table 1.

## 3. Results and Discussions

### 3.1. Orientation-Dependent Etching Rate Modeling

**Figure 4:**
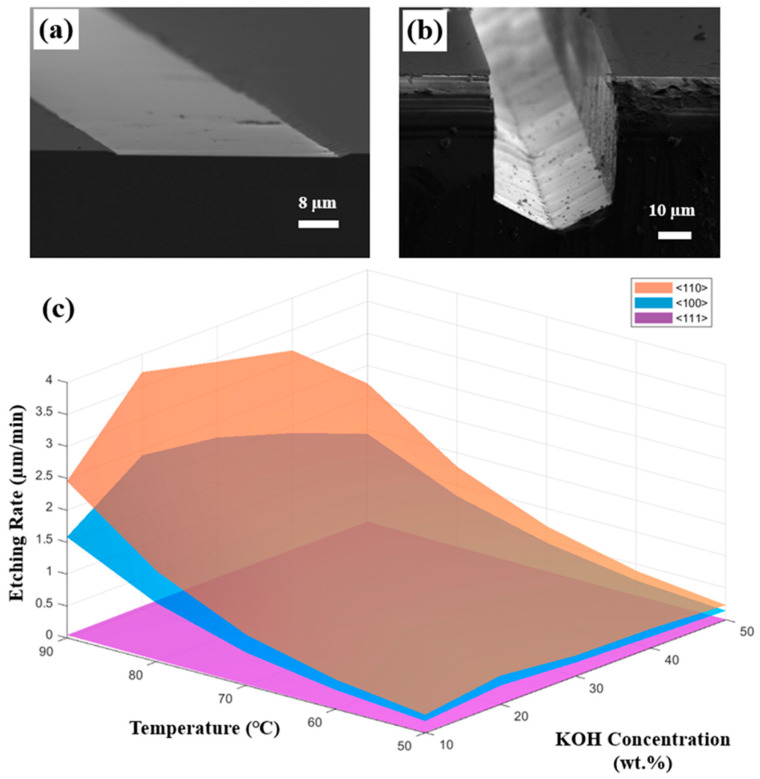
Cross-sections of Si wafers (**a**) before and (**b**) after etching; (**c**) summary of etching rates versus KOH concentration and temperature.

To quantify the orientation-dependent etching kinetics of (110) Si, the etching rates of the principal crystallographic planes {110}, {100} and {111} were systematically investigated at different concentrations and temperatures. Representative cross-sectional SEM images of the Si wafer before and after etching are presented in Figure 4a,b, illustrating the morphological evolution used for etching rate quantification.

The correlation between etching rates, KOH concentration and temperature was summarized in Figure 4c. It shows that increasing the temperature from 50 °C to 90 °C results in an exponential rise in etching rate for all orientations. For instance, at a concentration of 30 wt.%, the etching rate of the fast-etching {110} plane surges from 0.322 µm/min at 50 °C to 3.441 µm/min at 90 °C, which is a more than tenfold increase. The exponential influence of temperature indicates that etching is a thermally activated process. Furthermore, the data shows a consistent anisotropic trend in the etching rates along different orientations ($R_{hkl}$), $R_{110}$ > $R_{100}$ ≫ $R_{111}$. The highest removal rate was 3.720 µm/min at temperature of 90 °C in 20 wt.% KOH solution along {110} plane, while the {111} plane remains kinetically inert with rates consistently two orders of magnitude lower (0.005–0.062 µm/min).

Conversely, the dependence on KOH concentration is non-monotonic. Specifically, for the fast-etching {110} and {100} planes, the etching rate initially increased with concentration, reaching a peak at approximately 20–30 wt.%. However, with the further increase in KOH concentration beyond 30 wt.%, a continuous and significant rate reduction was observed across all tested temperatures.

To quantitatively describe the dependence of etching rate on temperature, KOH concentration and crystal orientations, a modified Arrhenius model was proposed. To introduce the influences of both temperatures and KOH concentration, a power-law concentration term was adopted to capture the combined effects of thermal activation and reactant availability. However, a simple power-law term (R ∝ $C^{n}$) fails to describe the rate reduction observed at high concentrations (>30 wt.%). Physically, the etching reaction requires not only hydroxide ions (${OH}^{-}$) for surface oxidation but also free water molecules ${(H}_{2}O$) for the solvation and transport of reaction products. At high KOH concentrations, the scarcity of free water becomes the rate-limiting factor [31,32]. To address both the effects of ${OH}^{-}$ and $H_{2}O$ supply across the entire concentration range, two concentration terms were included in the modified model. For different crystal orientation i (i = (100), (110), (111)), the etching rate $R_{i}(T,C)$ is expressed as:

(7) $$R_{i}\left(T,C\right)=A_{i}C^{n}{\left(1-C\right)}^{m}\exp\left(-\frac{E_{a,i}}{k_{B}T}\right)$$

where $R_{i}$ is the etching rate (µm/min), $A_{i}$ is the pre-exponential factor, C is the dimensionless mass fraction of KOH in aqueous solution (ranging from 0 to 1; e.g., 10 wt.% KOH corresponds to C = 0.1), (1 − C) represents the concentration of available water molecules ${(H}_{2}O$), T is the absolute temperature (K) and $k_{B}$ is the Boltzmann constant ($8.617\times 10^{-5}$ eV/K), $E_{a,i}$ is the apparent activation energy (eV), and n and m are the reaction orders with respect to hydroxide and water, respectively.

Nonlinear least-squares fitting was implemented based on Equation (7) using the Levenberg–Marquardt algorithm. Initial estimates for the parameters were determined via linear regression of the logarithmic transformation of Equation (7). To ensure the statistical reliability of the model, 95% confidence intervals were calculated for all fitted parameters. The fitted parameters and their corresponding coefficients of determination ($R^{2}$) are summarized in Table 2.

Table 2 reveals a significant crystallographic disparity in activation energy ($E_{a}$). For instance, the activation energy of etching along {111} plane is 0.6263 eV, which is notably higher than those of {110} and {100} planes. This fundamentally originates from the distinct atomic configurations of the Si surfaces. According to the classical atomistic etching model, the removal of a surface Si atom requires the breaking of its covalent bonds with the bulk lattice, known as back-bonds. An atom on the {111} surface possesses three back-bonds and only one dangling bond, whereas atoms on the {100} and {110} surfaces have 2 back-bonds and more dangling bonds, making them prone to nucleophilic attack by hydroxide ions. Consequently, the energy barrier for the {111} plane in etching is significantly higher.

On the other hand, the parameters in Table 2 also provide insight into the rate-limiting mechanisms. The reaction order of fast-etching planes is close to 1.0 (n ≈ 1.0), indicating a generally linear dependence on the ${OH}^{-}$ supply in the low-concentration regime. However, the high solvation order (m ≈ 3.0) implies that the etching process is heavily reliant on water molecules for product removal, which leads to the sharp decline in etching rate at high concentrations. In contrast, the {111} plane demonstrates much lower sensitivity to both species (n ≈ 0.5, m ≈ 1.3), showing its kinetic stability against concentration fluctuations.

The morphological evolution of (110) Si trenches is fundamentally governed by the kinetic competition between the fast-etching planes ({110}/{100}) and the slow-etching {111} sidewalls. To quantify this competition, an anisotropy factor γ was defined as the ratio of the etching rate:

(8) $$\gamma =\frac{V_{lat}}{V_{vert}}\approx \frac{R_{111}}{R_{110}}$$

By substituting the modified Arrhenius model (Equation (7)) and the kinetic parameters from Table 2 into Equation (8), the analytical expression for γ as a function of KOH concentration (C) and temperature (T) can be explicitly derived as:

(9) $$\gamma \left(T,C\right)\approx {\frac{A_{111}}{A_{110}}C}^{\left(n_{111}-n_{110}\right)}{\left(1-C\right)}^{\left(m_{111}-m_{110}\right)}\exp\left(-\frac{E_{a,111}-E_{a,110}}{k_{B}T}\right)$$

As indicated by this equation, since the etching rate of the fast-etching planes is significantly higher than those of the slow-etching planes, the anisotropy factor γ is highly sensitive to KOH concentration.

In the low-concentration regime (e.g., 10 wt.%), the reduced supply of ${OH}^{-}$ disproportionately decelerates the etching along fast-etching planes while having a minimal impact on the stable {111} planes. Therefore, the lateral convergence is effectively suppressed, which prevents the formation of V-shaped footing profiles.

Conversely, at high concentrations (e.g., 50 wt.%), although the absolute etching rates decreased due to water scarcity, this lack of free water disproportionately suppresses the fast-etching planes because of their strictly higher solvation dependence (m ≈ 3.0 for {110} and m ≈ 1.3 for {111}). According to Equation (9), this severe crystallographic disparity in water sensitivity inherently drives up the anisotropy factor γ, thereby exacerbating the V-shaped footing profiles.

Therefore, the derived analytical expression (Equation (9)) mathematically identifies low KOH concentration (10 wt.%) as the critical condition to reduce γ. This theoretical prediction provides the mechanistic basis for the defect-free morphologies. Having established the kinetic model and extracted the orientation-dependent parameters (Table 2), these quantitative data were subsequently incorporated into an ALE simulation to dynamically predict the morphological evolution of the trenches.

### 3.2. Simulation-Based Mechanism Analysis

Within the ALE formulation, the Si–solution interface is discretized into dynamically evolving mesh nodes. At each time step, the local crystallographic orientation (θ) of every surface element is evaluated, and the corresponding activation energy $E_{a}(\theta$) are assigned together with the reaction orders (n, m) according to the calibrated kinetic model in Table 2 [33,34]. The local normal etch velocity is then directly calculated using the modified Arrhenius model (Equation (7)). This orientation-resolved velocity field governs the time-dependent evolution of the trench geometry.

**Figure 5:**
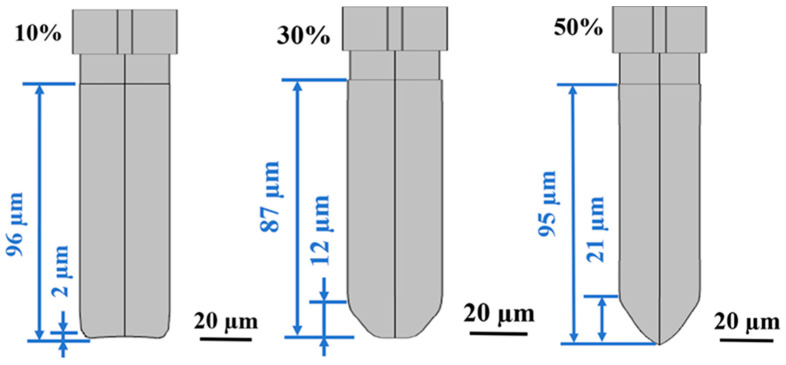
Theoretical prediction of geometric evolution and the critical flat-bottom depth at different KOH concentrations (10%, 30%, 50%).

As revealed by the simulated profiles (Figure 5), the trench morphology on (110) Si is governed by a geometric competition between two coupled velocity components: the vertical advancement ($V_{vert}$) and the lateral convergence ($V_{lat}$).

From this velocity competition, a deterministic geometric threshold naturally emerges, defined as the Critical Flat-Bottom Depth $D_{crit}$. This quantity represents the maximum trench depth attainable before the two opposing {111} sidewalls intersect at the bottom center.

The time of intersection can be calculated as:

(10) $$t^{*}=\frac{W_{mask}}{2\cdot v_{lat}}$$

where $t^{*}$ is the critical closure time required for the two opposite {111} sidewalls to intersect at the bottom, $W_{mask}$ represents the initial width of the mask opening, and $v_{lat}$ denotes the lateral convergence velocity of the {111} planes.

The corresponding critical flat-bottom depth is the vertical trench depth achieved at the critical closure time $t^{*}$. Combined with the definition of unified anisotropy factor γ, $D_{crit}$ can be defined as follows:

(11) $$D_{crit}=v_{vert}\cdot t^{*}=\frac{W_{mask}}{2}\cdot \frac{v_{vert}}{v_{lat}}=\frac{W_{mask}}{2\gamma }$$

In accordance with Equation (11), the simulated profiles under different KOH concentrations serve as a theoretical geometric baseline (Figure 5), explicitly demonstrating the progressive variation of this kinetic competition and its direct impact on the critical flat-bottom depth.

As calculated by the theoretical model (Equation (9)) and visually evidenced by Figure 5, at 10 wt.% KOH, the lateral convergence ($v_{lat}$) remains significantly smaller than the vertical advancement ($v_{vert}$), indicating a strongly anisotropic condition (γ ≪ 1). Consequently, the predicted critical depth $D_{crit}$ (740 µm) substantially exceeds the target trench depth (96 µm), ensuring that the sidewalls remain effectively parallel throughout the etching process. This geometric margin guarantees the formation of deep trenches with vertical sidewalls and flat bottoms.

At 50 wt.% KOH, the simulation predicts a pronounced decrease in kinetic contrast between crystallographic planes. Driven by this reduced anisotropy, the effective lateral velocity ($v_{lat}$) increases significantly relative to the vertical propagation rate ($v_{vert}$). As extracted directly from the ALE simulation (Figure 5), this accelerated lateral convergence causes the two {111} facets to intersect prematurely, forming a severe V-shaped profile with a footing height of 27 µm. This simulated morphological evolution aligns precisely with the theoretical trend predicted by Equation (11), confirming that the intersection occurs significantly below the target trench depth. Consequently, the results demonstrate that the V-shaped footing defect is not an anomalous artifact, but a deterministic geometric manifestation governed fundamentally by the kinetic competition ratio (γ).

The theoretical simulations strongly suggest that the footing defect is governed by the kinetic competition ratio γ, which is highly sensitive to process conditions. To validate these morphological predictions and determine the most favorable manufacturing window within the tested conditions, systematic experimental verifications were subsequently conducted.

### 3.3. Experimental Validation and Process Window Evaluation

**Figure 6:**
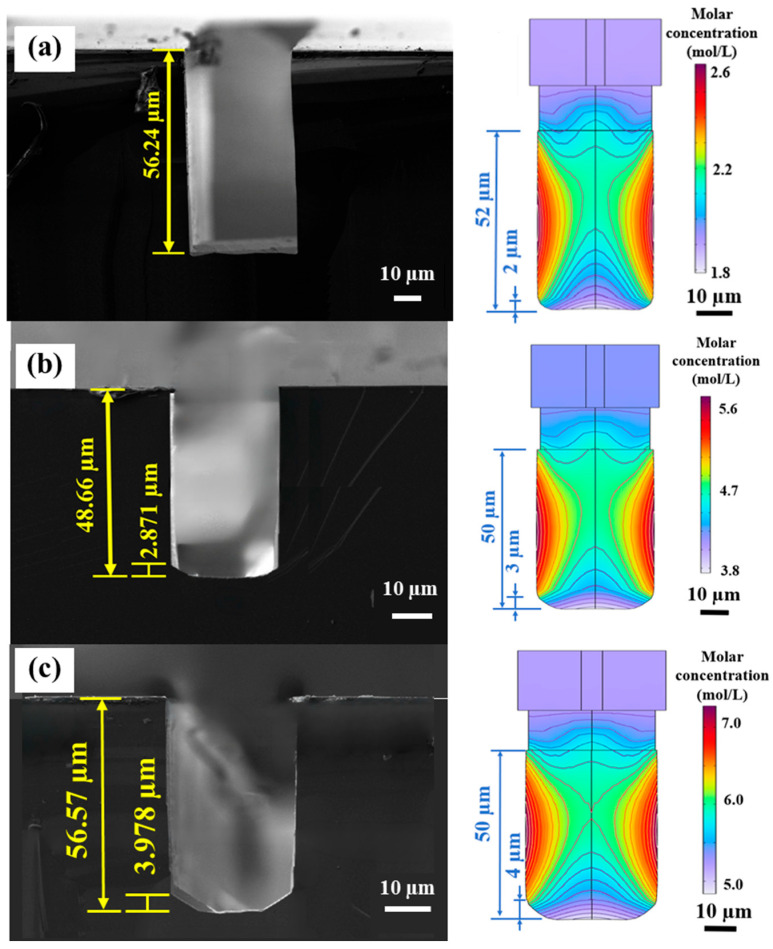

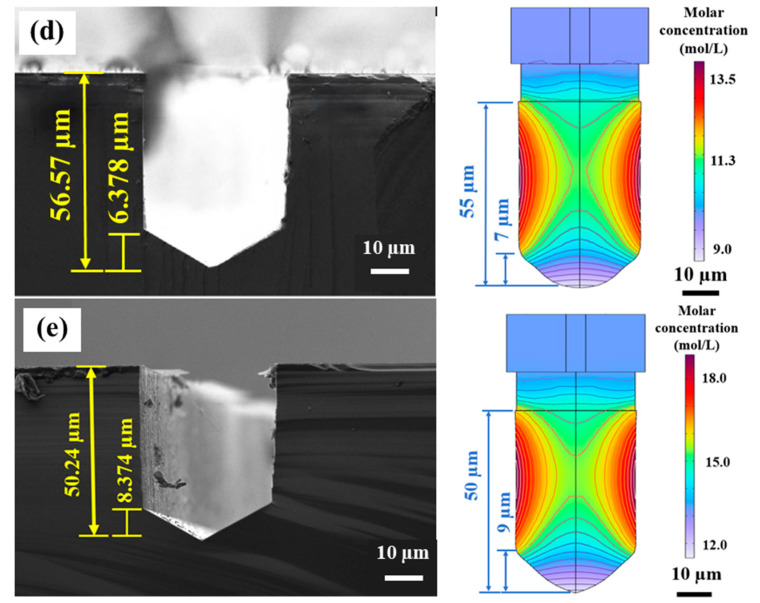
Comparison of representative cross-sectional SEM images (**left**) and corresponding ALE-simulated molar concentration distributions (**right**) for (110) Si trenches etched at 70 °C with different KOH concentrations: (**a**) 10 wt.%, (**b**) 20 wt.%, (**c**) 30 wt.%, (**d**) 40 wt.%, and (**e**) 50 wt.%.

Serial experiments were carried out to validate the simulation results. The morphological evolution of (110) Si trenches is highly sensitive to KOH concentration. To directly validate the theoretical predictions, the ALE-simulated molar concentration fields and the corresponding experimental SEM profiles at 70 °C are compared side-by-side in Figure 6. For all cases in Figure 6, the designed etching window width was fixed at 40 μm, and only the KOH concentration was varied. In the revised figure, the experimental and simulated panels are presented at the same physical scale to enable direct visual comparison. As shown in Figure 6, the geometric transition of the trench bottom can be clearly identified in both the simulated and experimental results as the concentration increased from 10 wt.% to 50 wt.%.

At a concentration of 10 wt.%, both the experiment and simulation (Figure 6a) demonstrate an ideal profile with a flat bottom and vertical sidewalls. As quantitatively compared in Table 3, the experimental footing profile is completely eliminated (0%), which closely matches the extremely low simulated value (3.85%).

**Figure 7:**
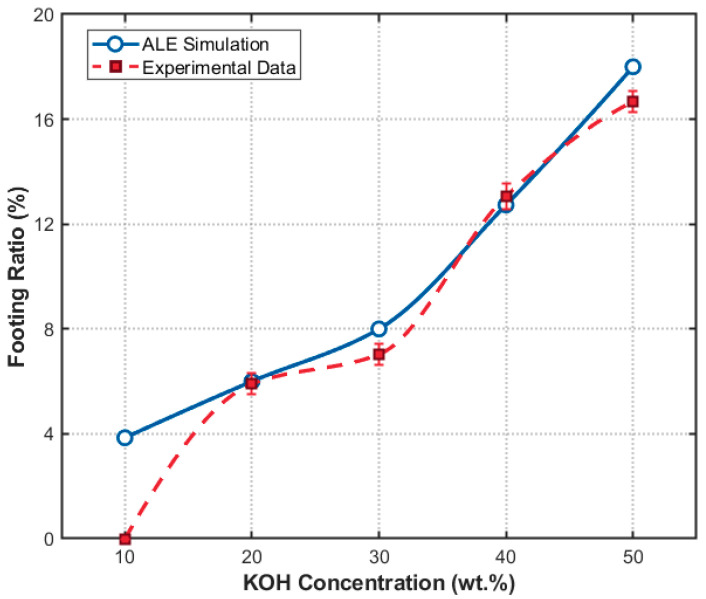
Variation of footing ratio with KOH concentration at 70 °C.

Conversely, increasing the concentration significantly exacerbates footing profile formation. As visually mapped by the multiphysics simulations and quantitatively compared in Figure 6 and Figure 7, and Table 3, the footing ratio exhibits a non-linear monotonic increase with concentration. At 50 wt.%, the experimental footing ratio reaches 16.67%, showing good agreement in the overall trend with the simulated prediction of 18.00%. In high-concentration solutions, although the overall etching kinetics are suppressed by the reduced availability of free water, the relative kinetic disparity between the fast-etching vicinal {110}/{100} planes and the slow-etching {111} planes still promotes lateral convergence and aggravates footing formation. This consistency between the ALE predictions and the actual etch profiles supports the proposed kinetic interpretation. Although solutions at 20–30 wt.% provide a higher etching rate (Table 3), they failed to maintain the structural fidelity required for high-precision MEMS devices.

**Figure 8:**
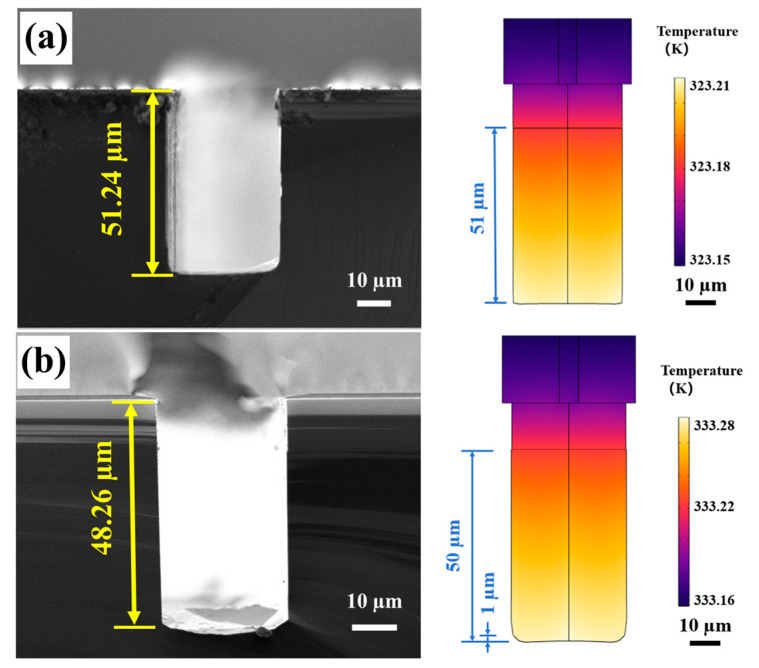

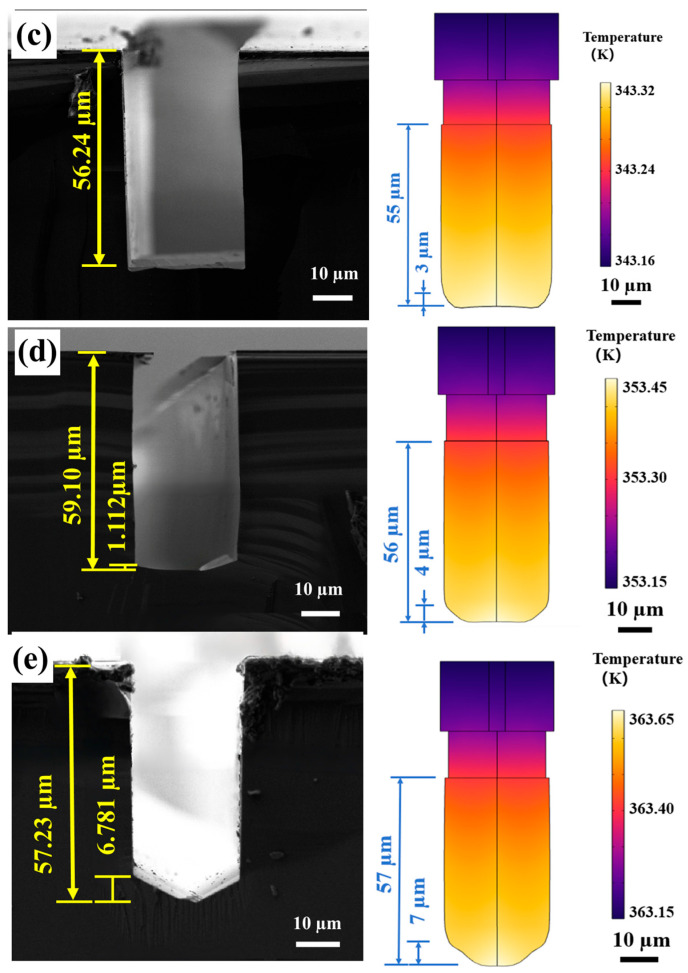
Comparison of representative cross-sectional SEM images (**left**) and corresponding ALE-simulated temperature distributions (**right**) for (110) Si trenches etched in 10 wt.% KOH solution at varying temperatures: (**a**) 50 °C, (**b**) 60 °C, (**c**) 70 °C, (**d**) 80 °C, and (**e**) 90 °C.

**Figure 9:**
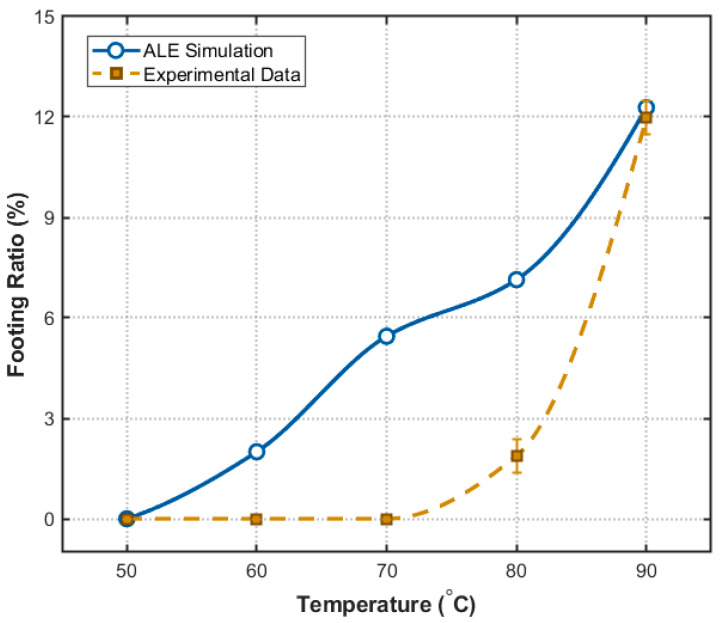
Variation of footing ratio with solution temperature at 10 wt.% KOH.

To directly validate the temperature effects, the heat-transfer-coupled ALE simulations and the corresponding experimental SEM profiles are compared side-by-side in Figure 8. For all cases in Figure 8, the designed etching window width was fixed at 40 μm, and only the etching temperature was varied. In the revised figure, the experimental and simulated panels are presented at the same physical scale to enable direct visual comparison. As dynamically mapped by the simulated temperature fields, the kinetic competition shifts significantly with rising temperature. The major geometric parameters are summarized in Table 4, and the corresponding quantitative trends are presented in Figure 9. The error bars in Figure 7 and Figure 9 represent the standard deviation obtained from three repeated experiments.

At lower temperatures (50–60 °C), both the experiment (Figure 8, left) and simulation (Figure 8, right) demonstrate that the kinetic competition favors high anisotropy. As quantitatively detailed in Table 4, the V-shaped footing profiles are entirely suppressed in the actual experiments (0%), remaining consistent with the minimal footing ratios predicted by the simulations (0–2.00%). However, this morphological advantage is offset by a prohibitively low etching rate. For instance, at 50 °C, 2 h etching is required to achieve a trench with a depth of 50 µm. This makes it inappropriate for high-throughput manufacturing.

As the temperature rises to 80–90 °C, although the etching rate increased exponentially, the footing ratio surges to over 10%. Specifically, at 90 °C, the experimental footing ratio reaches 11.85%, which is consistent with the simulated prediction of 12.28% (Table 4 and Figure 9). The ALE simulations successfully capture this macroscopic morphological degradation driven by the Arrhenius activation disparity (Figure 8d,e, right). However, while the simulation predicts relatively smooth sidewalls based on pure chemical kinetics, the actual experimental profiles at 90 °C (Figure 8e, left) reveal severe sidewall deterioration. This degradation is driven by two synergistic mechanisms. First, as extracted from our calibrated kinetic model (Table 2), the slow-etching {111} plane possesses a higher activation energy (0.626 eV) than the fast-etching {110} plane (0.572 eV). According to Arrhenius law, this theoretical disparity indicates that the reaction rate of the {111} plane is more sensitive to temperature elevation, promoting rapid lateral convergence of the {111} facets. On the other hand, the vigorous generation of hydrogen bubbles at extremely high temperatures induced a micro-masking effect and local turbulence, which disrupted surface passivation and further deteriorated the sidewall quality [35,36]. It should be noted that the present ALE model is deterministic and is mainly intended to capture the main dependence of trench depth and footing ratio on the process parameters within the investigated range, rather than to reproduce all local interfacial details under every condition. The error bars shown in Figure 7 and Figure 9 reflect experimental variability, whereas stochastic effects such as local bubble-induced micro-masking and sidewall roughening were not explicitly included in the present simulation.

Therefore, combining the competing requirements of morphological integrity, kinetic stability, and manufacturing throughput, 70 °C is identified as the most favorable temperature condition within the investigated range. At this temperature, the footing defect remains low while the etching rate is sufficient for practical fabrication.

**Figure 10:**
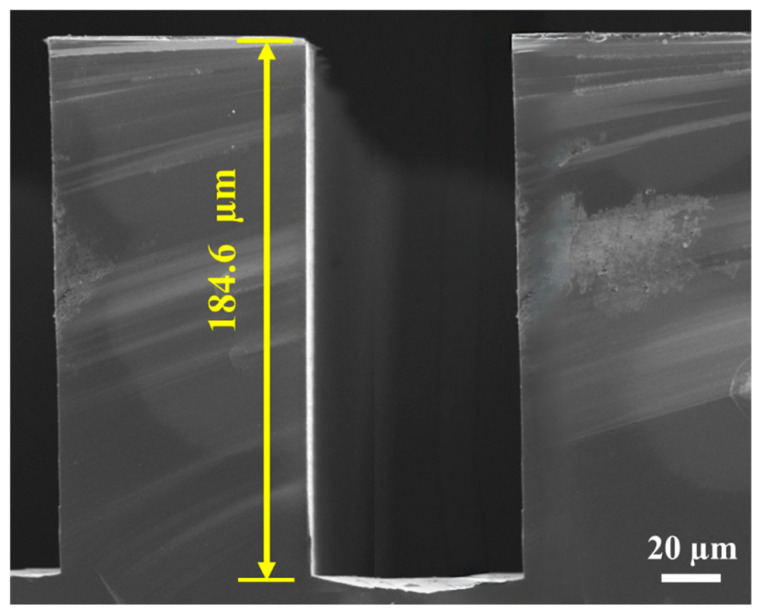
Cross-sectional SEM image of (110) Si trench etched under simulation-guided recommended conditions (10 wt.% KOH, 70 °C).

Based on the systematic evaluations above, combining a low KOH concentration with a moderate temperature provides the most favorable balance between etch efficiency and anisotropy. Among the investigated conditions, 10 wt.% KOH at 70 °C represents the most favorable process window within the tested conditions. To validate this, a complex high-aspect-ratio microstructure was fabricated. As shown in the representative SEM image (Figure 10), the trench depth reached an impressive 184.6 µm with almost no observable footing defects. The sidewalls remain nearly vertical and symmetric, which is consistent with the simulation predictions in terms of the overall morphology. This successful fabrication of ultra-deep complex features with no obvious V-shaped footing defects demonstrates the applicability of the proposed process condition for advanced MEMS manufacturing.

## 4. Conclusions

This study establishes a systematic framework coupling orientation-dependent Arrhenius kinetics with ALE-based multiphysics simulations to elucidate and suppress the footing profile formation in (110) Si anisotropic etching. The key conclusions are summarized as follows:

1. A modified Arrhenius kinetic model, incorporating dual concentration terms (hydroxide and free water), was established to quantify the etching rates of {110}, {100}, and {111} planes. The extracted parameters revealed that the slow-etching {111} plane possesses a notably higher activation energy ($E_{a}=0.6263\;eV$) compared to the {110} plane (0.5721 eV). This intrinsic thermodynamic disparity serves as the fundamental driver for the severe kinetic anisotropy.
2. The V-shaped footing defect is demonstrated to be a deterministic geometric manifestation governed by the kinetic competition ratio (γ). A Critical Flat-Bottom Depth ($D_{crit}$) was theoretically derived as a quantitative threshold for sidewall intersection. The 3D ALE-based multiphysics model, coupled with calibrated kinetics, was found to be consistent with the theoretical predictions in terms of the overall morphology evolution.
3. Systematic experiments showed that the experimental results generally followed the same trend as the simulations within the investigated parameter range. The footing ratio was found to increase monotonically with KOH concentration and temperature, confirming the trend predicted by the γ-based theoretical framework.
4. A dual-parameter kinetic regulation strategy was identified, with 10 wt.% KOH at 70 °C providing the most favorable process window within the investigated conditions. Under these conditions, ultra-deep and complex microstructures (depth reaching 184.6 µm) were successfully fabricated with almost no observable footing defect. This strategy provides a practical process reference for high-fidelity MEMS fabrication.

## Figures and Tables

**Table 1:** Parameters for Simulation model of Si wafer etching.

Parameters	Inlet $\mathrm{Velocity}\;v_{in}$ (m/s)	Outlet $\mathrm{Pressure}\;P_{out}$ (Pa)	$\mathrm{KOH}\;\mathrm{Concentration}\;C_{0}$ (wt.%)	$\mathrm{Temperature}\;T_{0}$	Simulation Time t (min)
Values	0.01	0	10, 20, 30, 40, 50	50, 60, 70, 80, 90	30

**Table 2:** Parameters for the {100}, {110} and {111} planes.

Orientation	Pre-Exponential Factor, ln(A)	Reaction Order ${OH}^{-}$, n	Solvation Order ($H_{2}O$), m	Activation Energy ($E_{a,i}$, eV)	Coefficient of Determination $R^{2}$
{110}	3.137 ± 0.082	1.018 ± 0.031	3.047 ± 0.095	0.5721 ± 0.012	0.9948
{100}	2.125 ± 0.094	1.035 ± 0.035	3.192 ± 0.102	0.5753 ± 0.014	0.9939
{111}	9.836 ± 0.150	0.512 ± 0.028	1.328 ± 0.067	0.6263 ± 0.018	0.9914

**Table 3:** Trench depth and footing ratio of (110) Si trenches at different KOH concentrations (70 °C).

KOH Conc.	10%	20%	30%	40%	50%
Trench depth (µm)	56.24	48.66	56.57	48.85	50.24
Footing height (µm)	0.000	2.871	3.978	6.378	8.374
Footing ratio (%)	0.000	5.900	7.032	13.06	16.67
Sim. Footing ratio (%)	3.846	6.000	8.000	12.73	18.00

**Table 4:** Trench depth and footing ratio of (110) Si trenches at different temperatures (10 wt.%).

Temperature	50 °C	60 °C	70 °C	80 °C	90 °C
Trench depth (µm)	51.24	48.26	56.24	59.10	57.23
Footing height (µm)	0.000	0.000	0.000	1.112	6.871
Footing ratio (%)	0.000	0.000	0.000	1.882	11.85
Sim. Footing ratio (%)	0.000	2.000	5.45	7.143	12.28

## Data Availability

Data is contained within the article.
